# Using Communities of Practice in the Home and Community-Based Service Workforce

**DOI:** 10.1093/geroni/igaf122.2342

**Published:** 2025-12-31

**Authors:** Sandi Lane, Caroline Yoon, Zavera Basrai, Yisel Pomier Maren, Trish Farnham, Kezia Scales, Gwen Tanner, Nathan Boucher

**Affiliations:** The University of North Carolina at Charlotte, Charlotte, North Carolina, United States; Duke University, Durham, North Carolina, United States; Duke University, Durham, North Carolina, United States; National Domestic Workers Alliance, Charlotte, North Carolina, United States; North Carolina Court of Appeals, Wilmington, North Carolina, United States; PHI, New York, New York, United States; Paraprofessional Healthcare Institute, New York, New York, United States; Duke University, Durham, North Carolina, United States

## Abstract

Older adults and people living with disabilities receive home- and community-based services (HCBS) from approximately 115,000 often inadequately supported direct service workers (DSW) in North Carolina (NC). The demand for these workers is increasing rapidly yet fragmented long-term services and support system (LTSS) deter new entrants into these careers. The previous stage of this Centers for Medicare/Medicaid Services (CMS)-funded project identified communication and relationship building as key competencies that DSW need to build career pathways, elevate skills and recognition for their work, and improve job quality and retention. Our project team then conducted a pilot DSW training program: asynchronous virtual training modules, virtual facilitated learning labs, and communities of practice (CoPs) led by direct service workers. The training program was designed to build knowledge and skills to support better communication with family caregivers through active listening, self-awareness, and self-management. A total of 50 DSWs from the categories of Home Health Aides, In-Home Aides providing limited assistance, personal care aides, direct support professionals, and self-directed direct care workers participated. Upon completion of asynchronous training, participants engaged in a two-hour learning lab facilitated by direct service workers followed by a series of DSW-facilitated peer learning groups called CoPs. These combined learning experiences encourage workers with lived experiences to engage in shared learning, peer support, and collective problem-based learning, which is crucial for reinforcing and contextualizing their knowledge leading to sustainable skills application in the workplace. We will discuss learnings from our process and the opportunity to scale the CoPs model for DSWs.

